# Stigma resistance among people with schizophrenia at Amanuel Mental Specialized Hospital Addis Ababa, Ethiopia: a cross-sectional institution based study

**DOI:** 10.1186/s12888-014-0259-y

**Published:** 2014-09-12

**Authors:** Berhanu Boru Bifftu, Berihun Assefa Dachew, Bewket Tadesse Tiruneh

**Affiliations:** College of Medicine and Health Sciences Department of Nursing, Gondar University, P.O. Box: 196, Gondar, Ethiopia; Institution of Public Health, Department of Epidemiology and Biostatistics, Gondar University, College of Medicine and Health Sciences, P.O. Box: 196, Gondar, Ethiopia

**Keywords:** Internalized stigma, Perceived stigma, Schizophrenia, Stigma resistance

## Abstract

**Background:**

Schizophrenia is one of the most disabling and severely stigmatized mental disorders. Together with social stigma, internalized stigma and perceived stigma can trigger a vicious cycle and diminishes the stigma resistance abilities of individual. Helping patients to cope up with perceived and internalized stigma play crucial role in fighting stigma. This study aimed to assess the prevalence and associated factors of stigma resistance among people with schizophrenia attending the outpatient department of Amanuel Mental Specialized Hospital, Addis Ababa, Ethiopia.

**Methods:**

Institution based cross-sectional study design was employed. Single population proportion formula was used to calculate sample size. Subjects were selected by systematic sampling techniques. Bivariate and multivariate logistic regressions were performed to identify the presence and strength of association. Odds ratios with 95% confidence interval were computed to determine the level of significance.

**Results:**

A total of 411 subjects participated in the study giving a response rate of 97.4%. The prevalence of low stigma resistance was found to be 51.6%. Rural residence (AOR = 0.29 (95% CI: 0.142, 0.594), difficulties of adherence to antipsychotic medication (AOR = AOR = 0.3, 95% CI: 0.155, 0.542), internalized stigma (AOR = 0.24, 95% CI: (0.111, 0.530), alienation (AOR = 0.5, 95% CI: (0.270, 0.927), stereotype endorsement (AOR = 0.37(95% CI: 0.312, 0.463) and social withdrawal (AOR = 0.27, 95% CI: (0.156, 0.468) were factors statistically associated with low stigma resistance.

**Conclusion:**

In this study, overall more than half of the study participants had low stigma resistance. Rural residence, difficulties of adherence to antipsychotic medication, high internalized stigma, alienation and social withdrawal were factors statistically associated with low stigma resistance. Encouraging participations in different social relationships such as befriending programs, family and peer support groups are recommended.

## Background

Schizophrenia is one of the most disabling and severely stigmatized mental disorder, which was selected as the central focus of the World Psychiatric Association’s global anti-stigma programme entitled ‘Open the Doors’ [1]. Stigma is common [2,3] and remains a major burden for individuals with psychiatric disorders [4,5], their families [6,7] and caregivers [8].

Stigmatizing attitudes towards people with mental illness such as: beliefs that people with mental illness are dangerous, cannot recover, or cannot contribute to society can lead them to internalize these stigmatizing beliefs and affects many domains of their lives such as self-esteem [9], prolong recovery [10,11] social relationships [10,12], treatment adherence and willingness to seek help [9], persistent suffering, disability and economic loss [13], difficulties of access housing and employment [14,15]. These consequence together with social stigma, internalized stigma and perceived stigma can trigger a vicious cycle and diminishes the stigma resistance abilities of individuals [14-16].

In order to overcome such problem, concerted efforts based on local evidence are required. A systematic literature review was conducted to identify stigma reduction strategies and interventions in the field of mental illness [16,17]. The review identified several levels at which interventions and strategies are being implemented. These are the intrapersonal (those focus on addressing internalized and anticipated stigma), interpersonal (those affecting intimate groups of people: friends, family, work and social networks), institutional (those that targeting the institutions particular relevance to stigmatized people: e.g. health care providers or police officers, community and governmental/structural are also involved in a variety of intervention such as training, education, media campaigns and contact people with mental illness, or combinations of these strategies [16-19].

Although a lot of work has been carried out on stigma by developing stigma reduction strategies, far less work has been done to assess the effectiveness of these stigma reduction strategies. The effective strategies identified mainly concentrated on the individual and the community level [18,19]. In order to reduce health-related stigma and discrimination significantly, single level and single target group approaches are not enough. Rather it required patient-centered approaches that target the intrapersonal level, to empower affected persons to assist the development and implementation of stigma reduction programs [20-22]. That is why the authors interested to identify the stigma resistance level of those patients.

Until a few years ago, the focus of most research was on investigation of stigma in people with schizophrenia through surveys of the general public’s attitudes and the stigma that people experienced after being diagnosed with a mental illness (internalized stigma) [16] or individual’s perceptions, or anticipation of negative social reaction (perceived stigma) rather than the stigma resistance ability of the patient. On the other hand, most of the researchers have been recommended to move on the alternative foci of research that is stigma resistance (SR). Stigma resistance is an individual’s capacity to counteract the stigma of mental illness [22,23].

Helping patients to cope with perceived and or internalized stigma especially building up of the stigma resistance ability of people with mental illness play crucial role in fighting stigma and help individuals in their hope of finding a fulfilling life in their recovery from mental illness and stigma because different literature revealed the importance of SR is positively associated with self-esteem, empowerment, and quality of life and negatively with stigma measures [23]. However, until now, stigma resistance has not been explicitly studied; the few available studies from Western demonstrated its importance. In developing countries, particularly in Ethiopia there is no previous study reported on stigma resistance of people with schizophrenia. The only published study concerning stigma among individuals with schizophrenia was internalized stigma [24]. Therefore, the present study aimed to assess the stigma resistance ability of individuals with schizophrenia and its associated factors.

## Methods

### Study design

Institution based cross-sectional study design was employed.

### Study area and period

The study was conducted from March to April 2012 at Amanuel Mental Specialized Hospital (AMSH). AMSH was established in 1930 and is situated in Addis Ababa, the capital city of Ethiopia. It is the only mental health hospital in the country. The hospital has a total of 300 beds of which 277 are for inpatients and 23 are Emergency beds. There is also a large out-patient service, with around 115,000 visiting outpatients department each year.

### Participants

The participants of this study were individuals with schizophrenia receiving follow-up care at the outpatient department of Amanuel Mental Health Specialized Hospital. Single population proportion formula (with the assumption of 5% margin of error, 95% confidence level and 50% proportion) was used to calculate sample size; and it was found to be 422 (including 10% non response rate). The total number of patients who visited the hospital for the last 12 months were taken from patient records and then the average number of patients per day calculated. Participants were selected by systematic random sampling technique. All individuals with a clinical diagnosis of schizophrenia coming for follow up with an age greater than or equal to 18 years were included. Individuals with schizophrenia who were unable to speak, hear and have no insight were excluded from the study.

### Instrument

Stigma resistance was investigated using the SR subscale of the internalized stigma of mental illness (ISMI) scale which was developed by Isomippoan [25] in close collaboration with members of the target population. ISMI is a four-point anchored Likert scale consisting of 29 items tool grouped into five subscales reflecting stigma resistance, alienation, stereotype endorsement, perceived discrimination and social withdrawal. The stigma resistance subscale, with five items, measures a person’s ability to resist or be unaffected by internalized stigma. The alienation subscale, with six items, measures the subjective experience of being less than a full member of society. The stereotype endorsement subscale, with seven items, measures the degree to which respondents agreed with common stereotypes about people with a mental illness. The discrimination experience subscale, with five items, measures respondents’ perceptions of the way they tend to be treated by others. The social withdrawal subscale, with six items, measures aspects of social withdrawal.

All items of ISMI were measured on a 4-point Likert-type agreement scale (1 = strongly disagree to 4 = strongly agree) were widely used across the world including Africa. The prevalence of SR was determined using the mean score of SR items greater than or equal to 2.5 as cutoff point for high stigma resistance and less than 2.5 for low SR. High internalized stigma defined on an item mean score of 2.5 or higher as cutoff point on 4 ISMI sub scales (SR subscale excluded). Prevalence of high alienation, stereotype endorsement, perceived discrimination and social withdrawal were also examined on their mean score of 2.5 or higher as cutoff point. This cutoff point was also used previously [23,25].

Furthermore, we asked two questions on antipsychotic medication adherence and difficulties of patients’ adherence to their clinic appointments (follow up). The antipsychotic medication adherence question asked about history of non-adherence with antipsychotic medications and whether the non adherence behavior was linked to low stigma resistance with yes/no response. Specifically asked questions “Have you ever discontinued your antipsychotic medication because of fear of stigma associated with your mental illness”? “Have you ever discontinued your clinic appointments (follow up) because of fear of stigma associated with your mental illness”?

### Data collection and analysis

Data were collected by face-to-face interview using pre-tested, semi-structured questionnaire consisting the socio-demographic, clinical and internalized stigma of mental illness scale questionnaires. The questionnaire translated into an Amharic and then translated back into English.

Data were coded and entered into EPI info version 3.5.3 statistical software and then exported to SPSS windows version 16 program for analysis. Descriptive statistics (frequencies, tables, percentages, means and standard deviation) were used for the socio-demographic and clinical variables including individual’s response to ISMI scale. Binary logistic regression and odds ratio with 95% confidence interval were used to identify the associated factors of stigma resistance. A significance level of 0.05 was taken as cut off value for all statistical significance tests.

### Ethical consideration

The study proposal was initially approved by the ethical review board of The University of Gondar and Amanuel Mental Specialized Hospital. A formal letter of permission was obtained from the hospital and submitted to the respective outpatient department. The information about the study was given to the participants. Written informed consent was sought for each participant who voluntary and fulfilled the inclusion criteria. Only anonymous data collected in private rooms.

## Results

A total of 411 participants participated in the study with 97.4% response rate. One of the participants failed to complete the interview because of the illness and nine questioners were not fulfilled properly.

### Socio-demographic characteristics

The majority of the participants were males 302 (73.5%). The mean (+SD) age was 33.24 (± 9.73 years. Two hundred and eighty nine (70.3%) of the participants were primary educated, 240 (58.4%) were Orthodox Christian followers and 286 (69.6%) were single in marital status. One hundred and six (35.5%) of the participants were Amhara in Ethnicity. Out of 411 participants, 257 (62.5%) were unemployed, 322 (78.3%) were living in urban areas and 360 (87.6%) were living with their families (Table 1).

**Table 1:** **Socio-demographic characteristics of participants (n = 411) at Amanuel Mental Specialized Hospital, 2012**

**Characteristics**	**Number**	**Percent**
**Sex**		
Male	302	73.5
Female	109	26.5
**Age**		
18-24	84	20.4
25-34	162	39.4
35-44	114	27.7
≥44	51	12.4
**Educational status**		
Can’t read and write	40	9.5
Primary	289	70.3
Secondary & above	52	20
**Religion**		
Muslim	91	22.1
Orthodox	240	58.4
Protestant	67	16.3
Catholic	13	3.2
**Marital status**	72	17.5
Married		
Single	286	69.6
Divorced/widowed	53	12.8
**Ethnicity**		
Oromo	132	32.1
Amhara	146	35.6
Gurage	98	23.8
Tigre	35	8.5
**Employment**		
Unemployed	257	62.5
Employed	154	37.5
**Residence**		
Rural	89	21.7
Urban	322	78.3
**Living arrangement**		
Family	360	87.6
Alone	51	12.4

### Clinical characteristics

Regarding the clinical characteristics of participants, 117 (28.5%) were treated for 2–5 years, 204 (49.6%) had ≤1 years duration of illness, 82 (20%) faced difficulties attending clinic, 64 (15.6%) were not adhere to their antipsychotic medication (Table 2).

**Table 2:** **Distribution of participants (n = 411) by their clinical characteristics at Amanuel Mental Specialized Hospital, 2012**

**Characteristics**	**Frequency**	**Percent**
**Duration of the treatment (in years)**		
≤1	101	24.5
2-5	117	28.5
6-10	90	21.9
>11	103	25.1
**Duration of illness (in years)**		
≤1	204	49.6
2-5	78	19
6-10	45	10.9
>11	84	20.5
**Difficulties of adherence to follow up**		
Yes	82	20
No	329	80
**Difficulties of adherence to antipsychotic medication**		
Yes	64	15.6
No	347	84.4

### Prevalence of stigma resistance

Overall, 212 (51.6%) of the study participants had low stigma resistance. Regarding each items of stigma resistance, the majority 382 (92.9%) of the participants were agreed or strongly agreed with the item “people with mental illness make important contributions to society” (Table 3). The overall mean internalized stigma and internalized stigma subscales were: internalized stigma 2.45, alienation 2.49, stereotype endorsement 2.36, discrimination experience 2.45, social withdrawal 2.48, and stigma resistance 2.52. Prevalence of high internalized stigma, high alienation, high stereotype endorsement, high experienced discrimination and high social withdrawal were found to be: 48.6, 57.7, 43.6, 45.7 and 52.3 respectively.

**Table 3:** **Distribution of participants (n = 411) by their response to internalized stigma of mental illness scale at Amanuel Mental Specialized Hospital, 2012**

**Items of ISMI subscale**	**Strongly disagree**	**Disagree**	**Agree**	**Strongly agree**
	**N (%)**	**N (%)**	**N (%)**	**N (%)**
**Alienation**				
I feel out of place in the world because I have a mental illness	18(4.4)	243(59.1)	141(34.3)	9(2.2)
I am embarrassed or ashamed that I have a mental illness	22(5.4)	171(41.6)	196(47.6)	22(5.4)
I feel inferior to others who don’t have a mental illness	5(1.2)	204(49.6)	181(44)	21(5.1)
I am disappointed in myself for having a mental illness	7(1.7)	172(41.8)	205(49.9)	27(6.6)
People without mental illness could not possibly understand me	7(1.7)	260(63.3)	131(31.9)	13(3.2)
Having a mental illness has spoiled my life.	4(1)	197(49.9)	169(41.1)	41(10)
**Stereotype endorsement**				
Stereotypes about the mentally ill apply to me	8(1.9)	96(23.4)	294(71.5)	13(3.2)
I can’t contribute anything to society because I have a mental illness	25(6.1)	341(83)	42(10.2)	3(0.7)
Because I have a mental illness, I need others to make most decisions for me	33(8)	298(72.5)	74(18)	6(1.5)
People can tell that I have a mental illness by the way I look	30(7.3)	202(49.1)	163(39.7)	16(3.9)
People with mental illness cannot live a good, rewarding life	14(3.4)	285(69.3)	98(23.8)	14(3.4)
Mentally ill people tend to be violent	5(1.2)	76(18.5)	309(75.2)	21(5.1)
Mentally ill people shouldn’t get married	68(16.5)	277(67.4)	62(15.1)	4(1)
**Discrimination experience**				
People often patronize me, or treat me like a child, just because I have a mental illness	30(7.3)	202(49.1)	170(41.4)	9(2.2)
People ignore me or take me less seriously just because I have a mental illness	18(4.4)	185(45)	193(47)	15(3.6)
Others think that I can’t achieve much in life because I have a mental illness	14(3.4)	156(38)	233(56.7)	8(1.9)
Nobody would be interested in getting close to me because I have a mental illness	20(4.9)	263(64)	122(29.7)	6(1.5)
People discriminate against me because I have a mental illness	15(3.6)	205(49.9)	163(39.7)	28(6.8)
**Social withdrawal**				
I stay away from social situations in order to protect my family or friends from embarrassment	11(2.7)	237(57.7)	158(38.4)	5(1.2)
Being around people who don’t have a mental illness makes me feel out of place or inadequate	16(3.9)	230(56)	160(38.9)	5(1.2)
I don’t socialize as much as I used to because my mental illness might make me look or behave “weird”	11(2.7)	184(44.8)	210(51.1)	6(1.5)
I avoid getting close to people who don’t have a mental illness to avoid rejection	9(2.2)	210(51.1)	189(46)	3(0.7)
I don’t talk about myself much because I don’t want to burden others with my mental illness	5(1.2)	185(45)	215(52.3)	6(1.5)
Negative stereotypes about mental illness keep me isolated from the “normal” world	10(2.4)	153(37.2)	218(53)	30(7.3)
**Stigma resistance**				
I feel comfortable being seen in public with an obviously mentally ill person	16(3.9)	161(39.2)	210(51.1)	24(5.8)
People with mental illness make important contributions to society	3(0.8)	26(6.4)	363(88.2)	19(4.6)
Living with mental illness has made me a tough survivor	12(2.9)	210(51.1)	176(42.8)	13(3.2)
In general, I am able to live my life the way I want to	26(6.3)	249(60.6)	129(31.4)	7(1.7)
I can have a good, fulfilling life, despite my mental illness	25(6.1)	244(59.4)	140(34)	2(0.5)

### Factors associated with stigma resistance

Bivariante and Multivariate regression analysis were performed to explore the association of socio-demographic, clinical and internalized stigma of mental illness with stigma resistance. From the bivariate analysis: residence, living arrangement, difficulties of adherence to antipsychotic medication, difficulties of adherence to follow up, internalized stigma, alienation, stereotype endorsement and social withdrawal were factors associated with low stigma resistance and entered in multivariate logistic regression for further analysis (Table 4).

**Table 4:** **Factors associated with stigma resistance (bivariate and multivariate) analysis, at Amanuel Mental Specialized Hospital, 2012**

**Explanatory variables**	**Stigma resistance**		**COR (95% CI)**	**AOR (95% CI)**	**p-value**
	**Hign N (%)**	**Low N (%)**			
**Residence**					0.001
Rural	33(8)	56(13.6)	0.55(0.342, 0.897)	0.29(0.142, 0.594)	
Urban	166(40.4)	156(38)	1	1	
**Living arrangement**					
With family	162(39.4)	198(48.2)	0.31(1.688, 6.182)	----	
Alone	37(9)	14(3.4)	1	1	
**Difficulties of adherence to follow up**					
Yes	51(12.5)	31(7.5)	2.01(0.303, 0.817)	----	
No	148(36)	181(44)	1	1	
**Difficulties of adherence to antipsychotic medication**					
Yes	40(9.7)	24(5.9)	1.97(0.293, 0.878)	0.3(0.155, 0.542)	<0.001
No	159(38.7)	188(45.7)	1	1	
**Internalized stigma**					
High	118(28.7)	81(19.7)	1.48(0.240, 0.534)	0.24(0.111, 0.530)	0.001
Low	105(25.6)	107(26)	1	1	
**Alienation**					
High	105(25.5)	132(32.1)	0.68(0.457, 1.003)	0.5(0.270, 0.927)	0.044
Low	94(22.9)	80(19.5)	1	1	
**Stereotype endorsement**					
High	85(20.7)	94(22.9)	0.94(0.456, 0.995)	0.37(0.312, 0.463)	0.006
Low	114(27.7)	118(28.7)	1	1	
**Social withdrawal**					
High	69(16.8)	146(35.5)	0.24(0.159, 0.362)	0.27(0.156, 0.468)	<0.001
Low	130(31.6)	66(16.1)	1	1	

From the multivariate analysis; rural residence (AOR = 0.29 (95% CI: 0.142, 0.594), difficulties of adherence to antipsychotic medication (AOR = AOR = 0.3, 95% CI: (0.155, 0.542)), internalized stigma (AOR = 0.24, 95% CI: (0.111, 0.530)), alienation (AOR = 0.5, 95% CI: (0.270, 0.927)), stereotype endorsement (AOR = 0.37 (95% CI: 0.312, 0.463)) and social withdrawal (AOR = 0.27, 95% CI: (0.156, 0.468)) were factors statistically associated with low stigma resistance (Table 4).

## Discussion

The aim of this study was to assess the prevalence and associated factors of stigma resistance among people with schizophrenia at Amanuel Mental Specialized Hospital. Overall, the prevalence of low stigma resistance was found to be 51.6%. This figure is lower than the study carried out in Europe that reported a prevalence rate of 67.5% [26]. This variation may be due to the fact that in this study all of the study participants were recruited from the outpatient department where as in the study reported in Europe less than half of the study participants were recruited from outpatient departments and the remaining study subjects were recruited from inpatient and day care clinic. This may indicate that outpatient individuals with schizophrenia may have higher stigma resistance than inpatient. The other possible explanations may be due to sample sizes difference, the population surveyed and the setting.

In contrast, the prevalence of low stigma resistance in this study (51.6%) is higher than the study reported by Sibitz et al. from Europe (36.7%) [23]. This variation may be due to in our study all of the study participants were confirmed schizophrenic patient who were on follow up, where as in the study reported by Sibitz et al. one third of the study participants were individuals with schizoaffective disorder. This may indicate that individual with schizoaffective disorder have higher stigma resistance than individual with schizophrenia. However, the prevalence of low stigma resistance in this study is similar with the study carried out across 14 Europe countries (50.8%) [27] and a systematic review reported by Gabriel et al. (47.4%) [28].

Even if the ways in which the participants’ experience of stigma resistance toward each items were different, a number of similarities were also reported. For example, from South Africa 84% of respondents agreed or strongly agreed with the item “people with mental illness make important contributions to society” compared with 92.9% in this study [29]. Similar results also reported from Tehran study toward each items of stigma resistance [30]. These similarities may indicate their similar effort to fight stigma.

Regarding the associated factors; those patients who participated in the study from rural residences were about seventy one times less likely to develop high stigma resistance, [AOR = 0.29 (95% CI: 0.142, 0.594)] than those patients who participated from urban residences. This may be due to the fact that people in urban areas were accessed to different types of stigma reduction strategies such as contact with the mentally ill patients, access for different teaching media such as news paper and television as compared to those lived in rural areas.

Those patients who had difficulties of adherence to their antipsychotic medication were about seventeen times less likely to develop high stigma resistance, [(AOR = 0.3, 95% CI: 0.155, 0.542)] than those patients who adhere to their antipsychotic medication. This may be due to fear of stigma and discrimination; patients may reduced their adherence to antipsychotic medication. Stigma may have impact upon adherence to antipsychotic medication through various psychological mechanisms such as loss of self-esteem and self-efficacy, demoralization, hopelessness and depression [9-12,23]. For example, almost half of all patients who were non-adherent to their medication attributed to discontinued because of stigma [24].

Those patients who experienced high internalized stigma were about seventy six times less likely to develop high stigma resistance, [(AOR = 0.24, 95% CI: (0.111, 0.530)] than those patients who experienced low internalized stigma. This is probably, since SR is a person’s ability to resist or be unaffected by internalized stigma [23,25,28] therefore; those patients may not build up their capacity to counteract the devaluation, shame, secrecy and withdrawal triggered by applying negative stereotypes to them in order to play crucial role in fighting stigma and help their hope of finding a fulfilling life in their recovery from mental illness and internalized stigma [23]. These findings support the evidence for the role of stigma resistance as an important indication to fight internalized stigma.

Those patients who experienced high alienation were about fifty times less likely to develop high stigma resistance, [(AOR = 0.5, 95% CI: (0.270, 0.927)] than those patients who experienced low alienation. This is probably, since alienation is the subjective experience of being less than a full member of society [25,28] therefore; those patients may simply accept what the society’s have in mind toward mental ill patients or they may not be psychologically ready to fight against negative attitude of the society toward them.

Those patients who experienced high stereotype endorsement were about sixty three times less likely to develop high stigma resistance, [AOR = 0.37 (95% CI: 0.31, 0.46)] than those patients who experienced low stereotype endorsement. This may be due to the fact that, since stereotype endorsement is occurs when stereotype agreement/when an individual endorses the common public stereotypes (e.g., people with mental illness are weak) and stereotype self-concurrence/when an individual applies the culturally internalized beliefs to him or herself (e.g., I am weak because I have a mental illness) [25]. This, in turn, yields decrements in self-esteem, self-efficacy and the stigma resistance of individuals. In the current study this can be evidenced by almost half of the study participants were agreed or strongly agreed with stereotype endorsement and more than half of the study participants had low stigma resistance.

Those patients who experienced high social withdrawal were about seventy three times less likely to develop high stigma resistance, [(AOR = 0.27, 95% CI: (0.156, 0.468)] than those patients who had low social withdrawal. This may be due to fear of stigma and discrimination, concealment of one’s illness was a concern of the respondents. In addition, withdrawal from social contacts, particularly by those patients who suspected negative attitude may practice as means of stigma reduction strategy.

### Strength of the study

This study is the first of its kind in Ethiopia that determined the stigma resistance of individual with schizophrenia.

### Limitations of the study

The lack of published literature in Ethiopia limits the discussion of the findings. Recall and response biases might have occurred when completing the questionnaire. Some important variables such as quality of life and severities of the symptoms were not assessed. In addition, some of the independent variables were assessed with single questions, for example treatment adherence and difficulties of follow up adherence to their clinic appointments that may lead some patients to respond improperly.

## Conclusion

In this study, more than half of the study participants had low stigma resistance. Rural residence, difficulties of adherence to antipsychotic medication, high internalized stigma, alienation and social withdrawal were factors statistically associated with low stigma resistance. These findings provide evidence for the role of stigma resistance as a viable target to fight internalized stigma and improve the treatment adherence of people with schizophrenia. Therefore participations in different social relationships such as befriending programs, family and peer support groups are recommended particularly more attentions are given for those coming from rural residence in the clinical care setting to improve their adherence to antipsychotic medication. Further researches with qualitative and quantitative study methods are also suggested, in order to explore the relation of socio-demographic and stigma resistance.
